# William Dove

**Published:** 1856-10-01

**Authors:** 


### Art. VI.—WILLIAM DOVE.

The much-agitated question respecting this man's moral and legal responsibility has been determinately and irrevocably settled by the public executioner! William Dove was hanged on the 8th of August. On the evening of the 1st of August the editor of this journal had a lengthened interview with Mr. Barret, Dove's solicitor, and Mr. Morley, one of the witnesses for the Crown. Mr. Morley was only called upon at the trial to depose to the fact of Mrs. Dove's death being caused by strychnine. No question was put to this witness respecting the prisoner's state of mind. Mr. Morley, however, entertaining a strong conviction of Dove's irresponsibility, considered it to be his duty, after his conviction, to cooperate with Mr. Barret and others in making strenuous efforts to save him from the gallows. With this humane object Mr. Morley, accompanied by Mr. Barret, came to London; and it was at this time that the discussion referred to in the subjoined letter took place. The letter explains itself.

23, Cavendish-square, Aug. 1st, 1856.

My dear Sir,—Since my interview with you and Mr. Morley, late on Friday night, I have given the subject of our earnest conversation and long discussion, much anxious thought and consideration.

You will recollect that at that interview I had no hesitation in expressing to you and Mr. Morley my decided and unqualified opinion respecting Dove's legal criminality. I have felt since his trial and conviction no sympathy for him, being strongly impressed with a notion that, if the punishment of death were under any circumstances justifiable, it should be carried into effect in Dove's case. I am bound, however, to confess, that after carefully and dispassionately weighing the additional facts laid before me by Mr. Morley and yourself illustrative of Dove's mental history, I have been induced somewhat to modify my opinion of the case. The words "defective intellect," embodied by the jury in their verdict, as justifying their recommendation of Dove to mercy, are not, according to my apprehension, accurately descriptive or expressive of Dove's mental condition.

His case is one of *imbecility*. If Dove's intellect were only "defective" or weak in the *popular* signification of these terms, he ought to be viewed as a responsible person. It would be fatal to the best interests of society if mere "defect of intellect" were considered in our courts of law as a valid excuse or plea in criminal cases. God forbid that so dangerous a doctrine should ever be propounded by those usually called upon to aid, by their scientific testimony, the administration of justice. If this doctrine be advanced, by what means are we to gauge the strength of the human intellect? Who is to decide upon the psychological test or standard of mental or legal responsibility in such cases? The great mass of criminals have admitted weak intellects, defective understandings, perverted moral sense, and no just recognition of the difference between *meum* and *tuum*. Such persons are, nevertheless, rightly considered as responsible for their actions, and are justly punished when they violate the law.

I think, however, the case of Dove may safely be removed from the category of healthy, sane, or even "weak"-minded men.

His conduct through life has been remarkably characteristic of imbecility or idiocy. It appears that his mental infirmity manifested itself in early life, and that those who were engaged in his educational training perceived a remarkable and obvious natural defect in the constitution of his intellect. His actions were not merely those of a wicked, vicious, or eccentric man, but they evidently sprung out of a stunted, irregularly developed, congenitally defective, and badly organized *brain* and *mind*.

If Dove had been made, a short period before murdering his wife, the subject of a commission of lunacy, the question at issue being his competency to *manage his property*, what, I ask, would have been the verdict of the jury? If the fact of his writing letters in blood to the devil, his faith in the supernatural power and predictions of Harrison, the wizard; the tremendous influence which this "weird" person obtained over him; his cruelties to animals; his having threatened to shoot his father, and afterwards himself; his cutting down his corn when quite green simply because his neighbour had cut his down when ripe; his recklessness of conduct, want of moral perception, his inability in early life to acquire ordinary knowledge, and other facts sworn to in evidence as illustrative of his sad mental state, were laid before an intelligent jury empannelled to try the question of Dove's mental soundness and ability to manage himself and his affairs, can any reasonable doubt be entertained as to the result of the inquiry? Upon evidence considerably less satisfactory and convincing than that adduced in Dove's case, I have seen juries unanimously decide as to the mental unsoundness of individuals. Consider, for example, the celebrated case of Mrs. Cumming. This lady was pronounced insane by a jury, among whom were several county magistrates, simply because she was fond of the society of a few favourite cats, had an impaired memory (no wonderful fact, considering her bodily indisposition and advanced age), and was alleged to entertain a strong aversion to some members of her family who had by force dragged her out of her own house, and confined her in a lunatic asylum. From my knowledge of the conduct of juries, I feel convinced that Dove never would have escaped the verdict of insanity if the question for their consideration had been of a *civil*, and not of a *criminal*, character—one of *property*, not of *life*.

If Dove's mind was so unsound as to render him manifestly incompetent to manage himself or his affairs, ought he to be viewed as altogether responsible for any criminal act he might be guilty of? I use the word "altogether" advisedly; for although I am willing to acquiesce in the wishes expressed by yourself, Mr. Morley, and others, to state in writing an opinion adverse to carrying into effect the *extreme penalty of the law* in Dove's case, *I am bound to say that I shall deeply regret if he were in consequence of his alleged "defect of intellect" to be exempt from punishment or penal servitude for the remainder of his life.* I have no hesitation, however, in asserting that it would be a great and fatal mistake and a grave fact of inhumanity to hang this wretched man. Considering the conclusive evidence of Dove's mental imbecility, his life, I think, ought not to be forfeited on the gallows.

The absence of all symptoms of delusion or hallucination renders the case different from those of ordinary insanity with which our Courts of law have to deal, and consequently to those not practically cognizant with the insane, the somewhat anomalous case of Dove appears one most difficult of comprehension. The eye of the practical psychological physician views in this case one of *modified responsibility*. As in many instances of indictment for capital crimes, the jury records, with the view of saving life, the merciful verdict of "manslaughter," instead of "murder," in consequence of the criminal having been impelled to the commission of the crime by great provocation, or been led to imbue his hands in the blood of a fellow-creature, during a moment of intense and uncontrollable mental irritation; so what is designated by lawyers as "partial" insanity, mental imbecility, or idiocy, when clearly and conclusively established, should invariably be considered as *greatly extenuating circumstances, or conditions of mind which should, in every case, absolve those so afflicted from the extreme penalty of the law.* I hope I have succeeded in clearly conveying to you and Mr. Morley my opinion of this deeply important case. If Dove were positively insane and quite incapable of distinguishing between right and wrong, I would send him to a lunatic asylum for the rest of his days, instead of to the hulks or to a penal settlement, but *recognising in his case a partial degree of responsibility, co-existing with much mental disorder, evidently interfering with the healthy exercise of thought, judgment, and volition,* it would be unjustifiable and cruel to treat him *like a perfectly sane man, or as an ordinary and responsible criminal, and consign him to the hands of the public executioner.*

I pray to God that so revolting an exhibition may not be witnessed, and that your humane and praiseworthy exertions to save this man's life may be crowned with success.

I remain, yours faithfully,

Forbes Winslow, M.D.

In justice to Mr. Morley, we append a copy of a letter in relation to W. Dove's state of mind, which he addressed to Mr. Baron Bramwell, the presiding judge.

Leeds, July 24, 1856.

My Lord,—The position in which I am placed in reference to the unfortunate man now under sentence of death, seems to make it my duty to address your lordship, although in so doing I feel that I must apologise.

The counsel for the crown, in his reply, commented on the fact, that I was not asked, by the defence, my opinion as to Dove's mental condition, and he argued from that omission that I, his medical attendant, must be of opinion that he was of perfectly sound mind.

In disclaiming the opinion thus attributed to me, I beg leave to submit to your lordship the view I really hold, and which, before the trial, I frankly expressed, to those engaged both for prosecution and defence.

The opinion I gave was to this effect—

1. That Dove possessed some knowledge of right and wrong, and was free from any dominant delusion which bore upon his crime, and that, therefore, he was not, in strict and legal sense, of insane mind.

2. That, nevertheless, his mind is so weak and disordered and morally helpless, as to give him only a minimum of self-control, leaving him powerless against evil influences and bad passions; and since diminished moral power must imply diminished responsibility, that his case is one requiring merciful consideration as to degree of punishment.

I do not attribute the weak and disordered state of Dove's mind merely to drunkenness, bad habits, or indulgence in vicious propensities. His mental peculiarities were evident long before these causes could be in operation. In his earliest infancy they were manifest. His mind had congenital defects, so that, as I believe, he never had the power which sane men possess to keep himself in a moral course of conduct.

This explains the fact that although bred in a family remarkable for goodness in its most pure and gentle forms, he alone, unlike every one else of the family, was a wicked, silly, and cruel child. It also explains the fact that although surrounded by the best examples, and supplied with every incentive to good, and placed under the care of excellent instructors, who tried every likely method of training, all these influences, contrary to ordinary experience, entirely failed, and left him as a man with the same mental weakness and disorder that had marked him when a child.

A principal defect in his mind appears to me to be its utter lack of moral sensibility; when he uses terms expressive of ideas of right and wrong, he has no corresponding feeling of their force and meaning, nor do such terms excite in his mind those emotions of a moral nature which they produce in a sound mind, and without which an intellect far higher than his would be powerless for good.

I can assure your lordship that this view of Dove's mental character is not set forth now for the first time, in order just to save his life. In one degree or another, it has always been the expressed opinion of those who knew him. He was from boyhood classed with those of weak and disordered mind. As proof that this is not an invention of the day, but an old and sincere opinion, I may state that I learn from intimate friends of the family, that his late father took him many years ago to persons familiar with the insane, for consultation as to the proper mode of treatment, and as to the propriety of restraint.

I enclose a copy of a letter alluded to on the trial, which was addressed to me during the inquest by a gentleman very competent to form an opinion, and of the highest integrity, but who, unfortunately, is just now in such an excitable state of mind, that it was not deemed safe to himself to subject him to the excitement of the witness-box.

I beg to apologise for the intrusion upon your lordship's attention,

And remain, my Lord,

Your Lordship's most obedient servant,

George Morley.

From earliest childhood to his committal to prison he was proved to have entertained one unbroken series of the wildest fancies that could have entered the mind of a person outside the walls of Bethlem, and to have been guilty, as a child, of extravagant, unnatural, and unaccountable conduct—such as chasing his sisters with a hot poker, setting fire to the curtains of his bedroom, locking up lighted candles in closets, tormenting cats and kittens; as a schoolboy, buying a pistol at twelve years old to shoot his father and schoolmaster, and convincing successive teachers at successive institutions of the perverse and irrational character of his mind, and of the impossibility of acting upon it by the ordinary means of educational treatment. His parents, in despair, tried the influences of agricultural teaching and employment. As a farmer's assistant he attempts to poison his master's horses—pours vitriol on his master's cows and calves. He hoists up the hinder parts of the cattle by ropes, threatens to murder his master, loads the musket of a fellow-servant till it bursts, delights in burning his master's hedges and cart-covers; he is never able to raise himself above the society of the farming boys, and at the end of five years is almost as ignorant of the science of husbandry as he was when he began. The effect of foreign travel is then tried. He is sent to Canada, not, as was suggested, as a sound man, able to take care of himself, but committed to the anxious care of friends in that country. He returns, and tells incredible stories of wild and imaginary adventures which he represents to have had with the American Indians. A small farm is then taken for him in his father's name, and shortly afterwards he marries. As a farmer and master of a family, he is continually setting fire to his housekeeper's cap, throwing water over her whilst ironing, recounting imaginary conflicts with robbers, patrolling the fields by night with fire-arms to meet imaginary assailants, leaping the hedges like a wild animal, riding and driving furiously, maltreating horses, killing trees by fantastical treatment, cutting his corn crops green, to be before his neighbours; attempting to fatten cows in a single night; incessantly playing with guns, pistols, and gunpowder, under the most dangerous circumstances, threatening the life of every one around him—his father, his mother-in-law, his wife, and himself; alternately quarrelling and gambolling with his wife, carrying her on his back, on his shoulders, in his arms and under his arm, amid the shouts of spectators; dealing with a wizard, sometimes for a spell upon the steward, sometimes for a spirit to torment his wife back to his bed. He fancies himself sold to the Devil, and his house haunted with strange noises. At Normanton, and afterwards at Leeds, he displays the same extravagance of conduct and delusion, and the circumstances of the crime itself, which are dwelt upon as proof of deliberate design, are pregnant with evidence of defective organization or permanent disease of the brain. He is actuated by a faith amounting to fanaticism in the predictions which he had received, or fancied he had received from the astrologer as to the term of his wife's existence.

He first asks the wizard for strychnine, then procures it in large quantities from the surgery of the very medical man who was attending his wife, and with the knowledge of at least two of the assistants!

He mentions the poison to his wife, his servant, and his neighbours. He summons old friends and new ones to the scene. He calls in his mother and sister, and writes for the mother and sister of his wife. On the first attack he runs for the surgeon's assistant, and betrays himself by asking whether there will be an examination of the body after death, and by protesting against it. He tells Mr. Morley, in his wife's presence, that she will die; and in defiance of assurances of her improvement, he insists to all comers on the certainty of her death; and shows his own sense of the fatality attached to the 1st of March by proclaiming, that if she gets over that day she will soon be well. When she is in her last struggles he runs for the surgeon and physician, and again objects on the road to a *post mortem* examination. Immediately after her death, he approaches the corpse of his wife, kisses it, exclaiming, "*So, Lord, teach us to number our days, that we may apply our hearts unto wisdom.*" During the inquest, he tells the astrologer in a public-house, overheard by several persons, that he had been dealing in strychnine, and in a second public-house shows how much strychnine would kill a person. Before and immediately after the death, he is constantly talking about marrying again, not merely to neighbours, servants, and absolute strangers, but to his deceased wife's own mother. It is suggested that his object was to marry Mrs. Whitham, a lady of irreproachable character and manners, who lived next door to him, and used to come into his house when summoned by rapping at the wall. Such being his presumed object, he invites a stranger, whom he had never seen in his life before, to come up with some boon companions, when he should see the wife's death announced in the newspapers, and have a regular jollification.

Dove's father, a man of exemplary excellence, entertaining strong suspicions of his insanity, consulted the governor of the county lunatic asylum as to the educational treatment proper to be adopted in his case. He accordingly applied every variety of means which science or experience could suggest for his mental treatment, short of confining him in a lunatic asylum. Recognising his state of mind, he cautioned the family of the deceased when his son had made proposals of marriage; and finally, by his last will, instead of providing for him as he did for his other children, he did so only by means of the trusts which it is usual to create for the protection of persons who are notoriously unable, in consequence of some mental unsoundness, to take care of themselves and property.

Such are the salient points with regard to Dove's mental condition, which we gather from the facts deposed to in his favour at the trial, as well as from Mr. Barret's able *résumé* of the case in his memorial to the Secretary of State and crown. We again repeat, that this man ought not to have been hanged. As a grave question had been raised as to his mental soundness and responsibility, and as the facts deposed to certainly established, at the least, a strong *primâ facie* case in the prisoner's favour, according to the first principles of our criminal code he ought to have had the benefit of this doubt, and to have been exempt from the extreme punishment awarded for his crime. We asked only that his life should be saved. Had we pleaded for his *exemption from all punishment*, we should have violated our own sense of public justice. In another part of the journal will be found Dove's final confession. This should be carefully read by all interested in his case. It throws great light upon his state of mind, and we think *conclusively establishes the justice of the line of defence adopted at the trial.* Dove was not a *lunatic* or an *insane* person, in the scientific signification of these terms; but there was just enough of congenital imbecility and defective intelligence in connexion with his state of mind as to justify the merciful recommendation of the jury in favour of a modification of punishment. If the Secretary of State, in deference to the advice of high legal authorities, considers that any good can result from hanging men like Dove, he will find, when it is too late, that he has committed an egregious blunder. Undue severity of punishment has no deterring or beneficial influence upon the criminal portion of the population. No fact in history is more conclusively established than this, *that severity of punishment invariably increases crime.* It is not our intention to discuss at any length the legal dicta of Mr. Baron Bramwell, as expounded in his charge to the jury. A few words will suffice for this part of the subject. Mr. Baron Bramwell declares that every man was presumed to be sane, until the contrary was proved to the satisfaction of the jury. "To establish a defence on the ground of insanity, it must be proved that he was, at the time he committed the offence, *labouring under such defect of reason, and such a disease of the mind, as not to know the nature of the act; or if he knew it, then that he did not know that what he was doing was wrong. If he did an act knowing it to be wrong, or contrary to the law of the land, he would be punishable. If he laboured under a partial delusion—if he was under the delusion that the deceased had inflicted some*

*injury upon him, and murdered her while under that delusion, he would be none the less amenable to punishment.* Therefore, according to the law as laid down by the highest authorities, to exempt a man from the penal consequences of his actions—those actions being contrary to law—the jury must be of opinion that at the time he did the act, he was not conscious that the act was one he ought not to commit. Of course that also meant that it was an act prohibited by law; because a man might imagine that there were some things perfectly right which were yet prohibited by law. For example, some persons might think it perfectly right to rob the rich to give to the poor; but if a person with such an opinion were to commit a theft, he would nevertheless be liable to the penal consequences of his acts. But if he not only thought it would be right so to do, but that he was not doing anything wrong or liable to punishment for it, then they should acquit him on the ground of insanity. Suppose a man imagined that another had done him an injury, and waylaid and murdered him, that imaginary injury would not be an excuse for the crime. But supposing the injury were real, and not imaginary, that would not justify him in taking away life; if he did so, he must suffer for it. Punishment was not merely administered in reference to an act done, but a criminal was punished to correct him and to hold out an example to others. If they punished a man who did not know he had done wrong, what example would that be to the world? People would say they had punished a man who was unconscious of wrong. But if they punished a man who possessed evil propensities and gratified them, they would deter him for the future, and also hold out a warning to others not to follow his example. If they let it go forth to the world that they would not punish a man who had a propensity or desire to do wrong, they would take away from persons in that position one of the things that would have prevented them from indulging in that propensity. Hence, to a man of weak mind and strong animal propensities, the knowledge that the law would not punish him would be to take from him one of the first and most powerful reasons for not repeating his crime."

According to the dicta of this Judge, no man is legally irresponsible unless he is in a state of absolute *dementia*, or, in other words, to use the language of Mr. Baron Bramwell, he labours "*under such a defect of reason, and such a disease of the mind, as not to know the nature of the act.*" In order to establish a valid plea of insanity in criminal cases, if this doctrine is to be held as orthodox, *a man must be proved to be idiotic or demented.* The great majority, or at least one-third of the insane actually in confinement at this moment, are, according to Mr. Justice Bramwell's exposition of the law, legally responsible for their actions, *for they are clearly conscious* of the "nature of their acts." It is a great mistake to imagine that persons decidedly insane, and undoubtedly irresponsible, have no knowledge of the "nature of their acts," and are not also fully cognisant that what they often do is both morally and legally "wrong."

We solemnly enter our protest against the doctrine that persons "partially insane," are to be viewed and treated as responsible beings! Science, justice, and humanity, cry out loudly against so fatal and monstrous a dogma. "Suppose," says Baron Bramwell, "a man *imagined* that another had done him an injury, and waylaid and murdered him, *that imaginary injury would not be an excuse for the crime.*"

We maintain, in the face of the world, and in the teeth of men of science practically acquainted with the phenomena of insanity, that it would be an iniquitous, unjustifiable, and an indefensible act to hang a man who committed murder under a delusion that the victim had "done him an imaginary injury." This was the character of M'Naughten's insanity: he shot Mr. Drummond under the influence of a delusion of this kind, and he was properly acquitted on the ground of insanity. Westron shot Mr. Waugh under somewhat similar circumstances, and his life was spared. In Westron's case there was some semblance of foundation for his impression relative to Mr. Waugh, although, not according to our judgment sufficient to justify his acquittal. The jury, however, recommended Westron to mercy on account of his "predisposition to insanity," and he escaped from the gallows. We presume that the insanity of this man is now *an acknowledged fact,* for we saw him not many days back, herding with some thirty or forty murderers in the criminal *den* of Bethlem Hospital! We feel that we are exposing ourselves to much obloquy, abuse, and animadversion for venturing to breathe a word against the strictly legal view of the question under consideration, raising a voice in favour of the criminal "partially insane," and going counter to popular clamour and prejudice. But we must not be deterred from an exposition of what we consider to be agreeable to the principles of SCIENCE, and in unison with the sacred cause of TRUTH, by any apprehensions like those referred to. All feelings of a personal character dwindle into utter nothingness when placed in juxtaposition with the great and eternal principles of justice and humanity. It is our duty, as public journalists, to expound what we conceive to be the right, philosophical, scientific, and practical view of the question of insanity in relation to jurisprudence, with the hope that the day is not far distant when men of great legal eminence will not only acknowledge the truth of our views, but act upon them when engaged in the solemn administration of the law.

